# Analysis of the volatile organic compounds of epidural analgesia-ameliorated metabolic disorder in pregnant women with gestational diabetes mellitus based on untargeted metabolomics

**DOI:** 10.3389/fendo.2023.1009888

**Published:** 2023-02-14

**Authors:** Si Ri Gu Leng Sana, Yang Lv, Guangmin Chen, Lei Guo, Enyou Li

**Affiliations:** Department of Anesthesiology, the First Affiliated Hospital of Harbin Medical University, Harbin, Heilongjiang, China

**Keywords:** gestational diabetes mellitus, volatile organic compounds, humoral biomarkers, epidural analgesia, solid-phase microextraction gas chromatography/mass spectrometry

## Abstract

Gestational diabetes mellitus (GDM) is a metabolic disease with an increasing annual incidence. Our previous observational study found that pregnant women with gestational diabetes had mild cognitive decline, which may be related to methylglyoxal (MGO). This study aimed to investigate whether labor pain aggravates the increase in MGO and explored the protective effect of epidural analgesia on metabolism in pregnant women with GDM based on solid-phase microextraction gas chromatography/mass spectrometry (SPME/GC-MS). Pregnant women with GDM were divided into a natural birth group (ND group, n = 30) and epidural analgesia group (PD group, n = 30). After fasting for ≥ 10 h overnight, venous blood samples were collected pre- and post-delivery to detect MGO, interleukin-6 (IL-6), and 8-epi-prostaglandin F2 alpha (8-iso-PGF2α) by ELISA. Serum samples were analyzed for volatile organic compounds (VOCs) using SPME-GC-MS. MGO, IL-6, and 8-iso-PGF2α levels in the ND group increased significantly post-delivery (*P* < 0.05) and were significantly higher in this group than the levels in the PD group (*P* < 0.05). Compared to the PD group, VOCs in the ND group increased significantly post-delivery. Further results indicated that propionic acid may be associated with metabolic disorders in pregnant women with GDM. Epidural analgesia can effectively improve the metabolism and immune function in pregnant women with GDM.

## 1 Introduction

With the implementation of the three-child policy in China, pregnancy complications have gradually increased. Gestational diabetes mellitus (GDM) is the most common complication (1). GDM can lead to maternal metabolic disorders and adversely affect the health of the mother and fetus (2, 3). In pregnant women with GDM labor pain stimulation during the perinatal period further aggravates metabolic disorders. Metabolomics is the study of biological systems, more specifically on investigating the changes in low molecular metabolites in the body after stimulation (4, 5). It mainly reflects the changes in endogenous metabolites caused by pathophysiological stimulation and disturbance. Metabolomics has great advantages in biomarker discovery, early disease diagnosis, pathogenesis research, and pharmacological and pharmacodynamic evaluations (6–12).

Childbirth is a physiological process that is accompanied by pain. labor pain is an unpleasant feeling due to the contraction of the uterus and transit of the fetus. Approximately 50% of the pregnant women regard labor pain as unbearable, which can instill fear (13). Labor pain can also be detrimental to the health of the child and the mother. Adverse effects on pregnant women and fetus include prolonged childbirth, uterine weakness, postpartum hemorrhage, fetal distress, and others (14, 15). labor pain can also lead to neuroendocrine changes in pregnant women.

Ding et al. (16) showed that the incidence of postpartum depression was as high as 34.6%, while epidural analgesia reduced the incidence of postpartum depression to 14.0%. The findings highlight the need for an analgesic method during delivery that does not affect the normal childbirth process, but which relieves pain. This innovation would be valuable for the physical and mental health of the mothers. An epidural analgesia technology is needed, especially for pregnant women with GDM. We have previously reported that GDM may cause memory loss in pregnant women (17, 18). labor pain may aggravate various GDM complications, including memory loss. This study investigated whether epidural analgesia technology in delivery can improve metabolism and serum methylglyoxal (MGO) and inflammatory, and oxidative stress factors in pregnant women with GDM.

## 2 Materials and methods

### 2.1 Subjects and protocol

Patients aged 18–35 years with American Society of Anesthesiologists physical status I-II were admitted to the study. Sixty pregnant women with GDM were selected and divided into either a natural delivery group (ND group, n = 30) or epidural analgesia group (painless delivery [PD] group, n = 30), according to whether the patient chooses labor analgesia. The study protocol was approved by the Ethics Committee of First Affiliated Hospital of Harbin Medical University and registered with the Chinese Clinical Trial Registry (registration number: ChiCTR2000038703).

Patients with pre-gestational type 1 or type 2 diabetes mellitus (T1DM and T2DM, respectively) were not included in the study. Patients with unnatural pregnancy or gestational periods <37 weeks or >41 weeks were excluded. Subjects taking medications, including corticosteroids, antidepressants, or antiepileptics, were also excluded. Additionally, subjects with chronic metabolic, endocrine, inflammatory diseases, cancer, drug or alcohol dependency, history of major brain abnormalities (e.g., tumors and hydrocephaly), epilepsy, or Parkinson’s disease were excluded. The psychological status of the pregnant women was assessed using the Hamilton Depression Scale. Those who scored > 7 and those who may have depression were excluded.

After the informed consent of the pregnant women, first of all, the pregnant women and their fetuses were monitored for oxygen inhalation and routine monitoring. At the beginning of the first stage of labor, when the uterine contraction is regular and the uterine orifice is opened to 2~3cm, let the pregnant woman take a lateral lying position, try to lower her head and hold her knees with both hands, conduct epidural puncture at the waist L2-L3 space, slowly inject the needle, place a tube 3cm to the head after the puncture is successful, and connect the syringe to draw back the bloodless and brain free spinal fluid, then inject 3 ml of 1.5% lidocaine into the epidural cavity. The epidural catheter was excluded from entering the blood vessel or subarachnoid space by mistake. When the vital signs of pregnant women are stable and there is no abnormality in the anesthesia level, the loaded dose of drug is 6 ml by epidural injection, and the drug is 0.1% ropivacaine+0.5 μg/mL sufentanil. Continuously observe the vital signs of pregnant women, and measure and maintain the anesthesia level at the T10 level. The continuous dose of the epidural link self-control analgesia pump is 4-6 mL/h, and the self-control dose is 2 ml. The administration will be stopped after the uterine orifice of the parturient is completely opened. During delivery, the two groups of pregnant women can drink sugar water and honey water to provide energy supply for pregnant women.

On the survey date, all the enrolled patients underwent routine medical history inquiries, physical examinations, and provided samples for laboratory measurements. The clinical research coordinators used a standard questionnaire to collect information on demographic characteristics and medical history. All pregnant women were instructed to maintain their usual physical activity and diet for at least three days before the survey. In order to evaluate the onset of analgesia, mothers’ pain was estimated using the Visual Analogue Score (VAS, 0: no pain 10: the worst pain) at analgesia request and at 20 minutes after administration of the initial bolus. After overnight fasting for ≥10 h, venous blood samples were collected to detect levels of glycosylated hemoglobin (HbA1c) and blood glucose (Glu). For each participant, blood was collected (3 mL) and centrifuged. The serum was recovered. Post-delivery, visual analog scale (VAS) scores were determined and the venous blood of each woman was collected and treated as just described. Blood samples were stored in a -80°C deep cryogenic refrigerator. MGO, interleukin-6 (IL-6), and 8-epi-prostaglandin F2 alpha (8-iso-pgf2α) were detected using ELISA. All measurements were performed within 6 months of sample collection.

### 2.2 Solid-phase microextraction

Post-delivery, the venous blood of the two groups was analyzed using SPME. In this study, we selected a 75 μM extraction head. The coating material was carbon molecular sieve/polydimethylsiloxane (car/PDMS). An automatic sample injector was used for heating and extraction. The liquid sample bottle was accessed *via* a puncture. An extraction method for headspace extraction was adopted. The SPME fiber was inserted into evacuated 20ml glass vials and exposed to the headspace of a blood sample (blood: 2 ml, taken from the ulnar vein) for 20 min at 40°C. After the extraction and concentration of the samples, the automatic sampling device inserted the extraction head into the gas chromatography-mass spectrometry (GC-MS) injection port for analysis. The desorption of volatiles occurred in a hot GC injector at 200°C for 2 min.

### 2.3 GC-MS analysis

All analyses were performed on a model QP2010 GC/MS (Shimadzu) equipped with a DB-5MS porous layer open tubular column (length: 30 m; internal diameter: 0.250 µm; film thickness, 0.25 mm: Agilent Technologies). The injections were performed in splitless mode, with a splitless time of 1 min. The injector temperature was set to 200°C and the carrier gas was helium at a flow rate of 2 mL/min. The temperature in the column was maintained at 40°C for 2 min to condense the hydrocarbons. The temperature was then increased to 200°C at 70°C/min and held for 1 min. Subsequently, the temperature was ramped to 230°C at a rate of 20°C/min and maintained for 3 min. MS analyses were performed in full-scan mode with an associated m/z range of 35–200 amu. An ionization energy of 70 eV was used for each measurement, and the ion source maintained at 200°C.

### 2.4 Statistical analyses

SPSS19.0 software was used for the statistical analyses. All data were tested for normality and variance. Normally distributed data are expressed as mean ± standard deviation (mean ± SD). Continuous variables with normal distribution were compared using Student’s t-test. Variables with abnormal distribution were compared using the Mann-Whitney U test. The least significant difference method was used to make multiple comparisons among the groups. Categorical data are expressed as counts and percentages, and comparisons between groups were performed by bilateral χ 2 inspection. *P* < 0.05 indicated statistical significance.

SIMCA-p +11.5 software was used for multivariate data analysis and model establishment. Principal component analysis (PCA) and partial least squares-discriminant analysis (PLS-DA) were used for statistical analysis. At the same time, the PLS-DA sample distribution score map was used to divide different samples into different clusters. The default seven-round cross-validation method was used, and the value of the variable importance in projection (VIP) of relevant variables in the PLS-DA model was calculated. Two hundred iterative permutation tests were conducted to verify the supervision mode and to prevent overfitting of the PLS-DA model. The nonparametric Kruskal–Wallis rank sum test was used to calculate the *P*-value. When VIP > 1.0, and *P* < 0.05, the difference variable was statistically significant (i.e., the metabolite was a significantly different metabolite).

## 3 Results

### 3.1 Demographic information

There were no significant differences in body weight, blood glucose level, or HbA1c level between the two groups (*P* > 0.05) (**Table 1**). The VAS score of the PD group was significantly better than that of the ND group *(P* < 0.05) (**Table 1**).

**Table 1:** Demographic characteristics.

	PD	ND	F	*P*
Sample	30	30		
Age, years	30.16 ± 3.27	30.79 ± 4.16	0.65	0.52
Height, cm	165.32 ± 2.89	164.91 ± 2.51	0.59	0.60
Weight, kg	79.36 ± 11.58	78.35 ± 13.57	0.31	0.76
Glucose, mmol/L HbA1c, % VAS	4.90 ± 1.32 5.31 ± 0.61 3.1 ± 0.7	5.39 ± 0.71 4.99 ± 0.93 7.8 ± 2.0	1.79 1.78 6.215	0.07 0.08 0.05

Vas, Visual Analogue Scale/Score; ND, Natural Delivery Group; PD, Epidural Analgesia Group; HbA1c, Glycosylated Hemoglobin.

### 3.2 Level of serum indicators

Pre-delivery, the levels of MGO, IL-6, and 8-iso-pgf2α in the ND group were not significantly higher than the levels in the PD group (*P* > 0.05). Compared to pre-delivery, the levels of MGO, IL-6, and 8-iso-pgf2α in the PD group post-delivery showed an upward trend, but the difference was not statistically significant. However, post-delivery, the levels of MGO, IL-6, and 8-iso-pgf2α in the ND group significantly increased (*P* < 0.05). These levels in the ND group post-delivery were also significantly higher than those in the PD group (*P* < 0.05) (**Table 2**).

**Table 2:** Comparison of prenatal and postpartum serum MGO, IL-6, and 8-iso-PGF2α levels between the PD and ND groups.

Group	n	MGO		*P*
		PD	ND	
Pernatal	30	46.15 ± 8.27	45.34 ± 7.79	0.69
Postpartum	30	67.34 ± 18.81	95.35 ± 18.27	<0.001
*P*		<0.001	<0.001	
Group	n	IL-6		
		PD	ND	*P*
Pernatal	30	28.31 ± 4.26	26.86 ± 4.01	0.18
Postpartum	30	35.51 ± 4.21	44.17 ± 5.51	<0.001
*P*		<0.001	<0.001	
Group	n	8-iso-PGF2α		
		PD	ND	*P*
Pernatal	30	47.13 ± 8.26	48.19 ± 7.97	0.61
Postpartum	30	61.24 ± 7.22	81.15 ± 6.98	<0.001
*P*		<0.001	<0.001	

ND, Natural Delivery Group; PD, Epidural Analgesia Group; MGO, methylglyoxal; IL-6, interleukin-6; 8-iso-pgf2α, 8-epi-prostaglandin F2 alpha.

### 3.3 Metabolic volatile organic compounds

**Figure 1:**
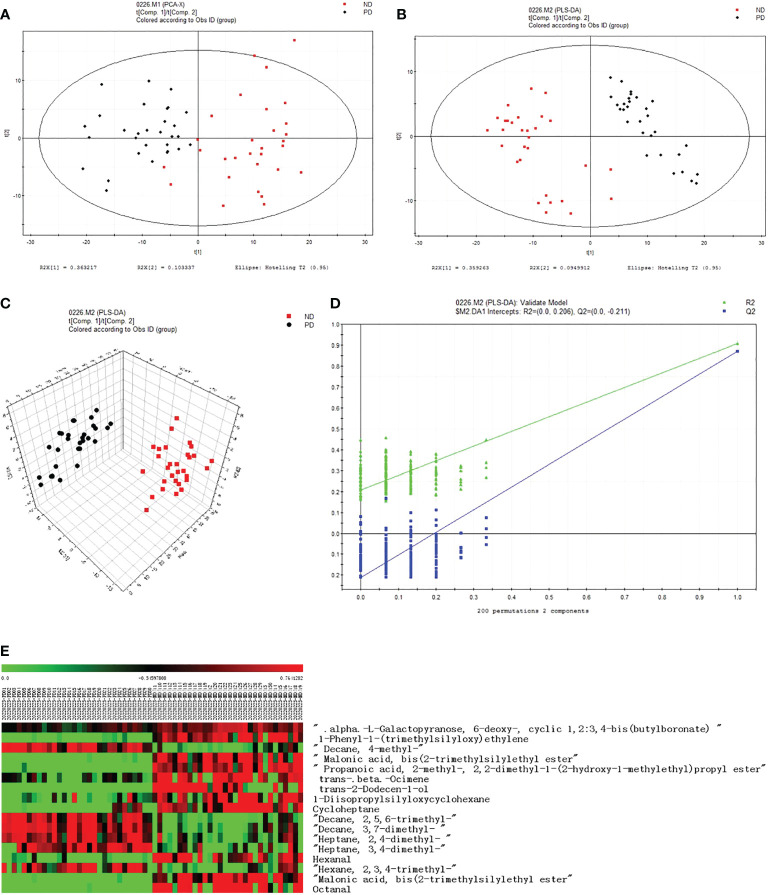
VOCs analysis chart. **(A)** PCA score plot, (R2X (1)=0.363217; R2X (2)=0.103337). The confidence level was the default of the software SIMCA-P 11.5, and its threshold was 95%. **(B)** PLSDA score plot, (R2X (1)=0.359263; R2X (2)=0.0949912). **(C)** PLS-DA score plot. **(D)** PLS-DA validation plot intercepts, R2=(0.0,0.206), Q2=(0.0,-0.211). **(E)** Heat map of two groups of different substances.

Compared to the PD group, the metabolic VOCs in the ND group increased significantly post-delivery. In this study, we found 17 different metabolites (**Table 3**). Of these, 11 were in the ND group and six were in the PD group. In the two-dimensional PCA score diagram, data from the two groups showed a good separation trend (**Figure 1A**). When using a single prediction component and three orthogonal components, the PLS-DA score chart displayed the data of the two groups and also had a good separation effect (**Figures 1B, C**). Two hundred iterations were performed to test the supervision model. The R2 and Q2 values calculated from the converted data were lower than their original verification values, which proved the effectiveness of the supervision model (**Figure 1D**). Based on the heat map (**Figure 1E**), the expression of six types of VOCs increased in the PD group as indicated by the red color. Eleven types of VOCs were increased in the ND group.

**Table 3:** Differential metabolites in blood VOCs of pregnant women with GDM.

VOCs	VIP	*P*	RT	FC (PD/ND)
Alpha-L-galactopyranose, 6-deoxy-, cyclic 1,2:3,4-bis(butylboronate)	1.33661	0.0000	10.69167	-0.191668188
1-Phenyl-1-(trimethylsilyloxy)ethylene	1.26562	0.0000	7.816667	-0.861086889
Decane, 4-methyl	1.40738	0.0000	8.991667	0.634477509
Malonic acid, bis(2-trimethylsilylethyl ester	1.29241	0.0000	18.10833	-0.953999982
Propanoic acid, 2-methyl-, 2,2-dimethyl-1-(2-hydroxy-1-methylethyl)propyl ester	1.31679	0.0000	15.91488	-1.001862334
Trans-beta-ocimene	1.07303	0.0003	6.916667	-0.424311265
Trans-2-dodecen-1-ol	1.12905	0.0000	12.3649	-1.13140546
1-Diisopropylsilyloxycyclohexane	1.13159	0.0000	13.10833	-0.483747132
Cycloheptane	1.24853	0.0000	6.3	-0.59981966
Decane, 2,5,6-trimethyl-	1.28815	0.0000	8.777957	0.709318109
Decane, 3,7-dimethyl-	1.42028	0.0000	9.725	0.561697272
Heptane, 2,4-dimethyl-	1.15657	0.0000	4.45	0.521234226
Heptane, 3,4-dimethyl-	1.08788	0.0000	3.491667	0.478836917
Hexanal	1.09592	0.0000	4.375	-0.829923822
Hexane, 2,3,4-trimethyl-	1.04946	0.0000	5.484654	0.504141371
Octanal	1.30959	0.0000	4.422312	-1.054138731

VIP, Variable importance in the projection; FC, Fold change; VOCs, volatile organic compounds.

## 4 Discussion

Severe labor pain makes pregnant women vulnerable to fear and anxiety, and heightens their stress. These feelings can be exacerbated in pregnant women who develop GDM. These stressors have several adverse effects on the mother and fetus. During delivery, the body is in a state of stress due to stimulations that include pain and childbirth trauma (19). The immune function of the body is disrupted, which increases the risk of maternal infection and other complications. In recent years, epidural analgesia has been used in many patients. An increasing number of pregnant women and obstetricians are accepting epidural analgesia, which has an obvious effect on reducing labor pain.

Under physiological conditions, a variety of enzymes participate in the metabolism of MGO. A small amount of MGO in the body is not sufficient to cause toxic reactions. However, metabolic disorders in patients with GDM lead to an increase in MGO production. Simultaneously, the strong stress response caused by delivery leads to an increase in levels of reactive oxygen species, which stimulate the production of MGO (20). In a previous study, we found that pregnant women with GDM were in a state of metabolic disorder (21). In the process of delivery, this metabolic disorder is aggravated because the delivery process itself can be considerably traumatic. In this study, the results are consistent with this phenomenon. The level of MGO in both groups increased post-delivery. However, compared with the PD group, the level of MGO in the ND group increased significantly, indicating that epidural analgesia can ameliorate metabolic disorders of MGO caused by labor pain.

Under physiological conditions, immune changes produced by delivery have protective significance for the body (22). However, abnormal immune function disorders are closely related to perinatal diseases. Studies have shown that labor pain is the main factor that causes changes in immune function (23–25). Epidural analgesia can effectively protect the body from excessive stress and inhibit immune function. IL-6 and 8-iso-pgf2α were also detected in this study. Compared with the PD group, the levels of IL-6 and 8-iso-pgf2α in the ND group post-delivery increased significantly, indicating that epidural analgesia can reduce the inflammatory and oxidative stress response of pregnant women with GDM.

This study mainly observed the effects of different delivery methods (ND and PD) on the metabolism of pregnant women with GDM. GC-MS was used in a novel analysis of the changes in VOCs in pregnant women with GDM post-delivery. In the two groups, we found 15 kinds of VOCs, Including 10 VOCs in the ND group and 5 VOCs in the PD group. The results revealed obvious metabolic disorders in the ND group. Ten differential substances were found in the ND group, including alpha-l-galactopyranose; 6-deoxy-, cyclic 1,2:3,4-bis(butylboronate); 1-phenyl-1-(trimethylsilyloxy)ethylene; malonic acid; bis(2-trimethylsilylethyl ester); propanoic acid; trans-beta-ocimene; trans-2-dodecen-1-ol; 1-diisopropylsilyloxycyclohexane; cycloheptane; hexanal; octanal。

Among the different substances in the ND group, alcohols (trans-2-dodecen-1-ol) and aldehydes (hexanal and octanal) were identified. Active aldehydes are mainly produced during lipid and glucose metabolism (including enzymatic and non-enzymatic pathways). The enzyme pathway is usually an aldehyde intermediate or by-product produced during glucose and lipid metabolism *in vivo* (26). This is also consistent with the disorder of active aldehyde metabolism observed in pregnant women with GDM. Under pathological conditions, aldehyde metabolism is disordered, resulting in abundant accumulation of aldehyde and formation of an aldehyde microenvironment (27). Similar to saturated aldehydes, hexanal is oxidized to the corresponding caboxylic acid by aldehyde dehydrogenase mainly in the liver, but also in other tissues and cells. The acid can serve as a substrate for the Krebs cycle or is excreted as a salt. Alternatively, it can conjugate with glutathione or the sulfhydryl group of other proteins. Free radical induced lipid peroxidation may play a role in neurodegeneration and peroxidation leads to the formation of hexanal from omega-6 fatty acids. We have previously demonstrated *in vitro* that pyruvate dehydrogenase (PDH) catalyzes the condensation of saturated aldehydes with pyruvate to form acyloins. We have further shown in perfused rat heart that hexanal, presumably *via* PDH, is converted to 3-hydroxyoctan-2-one and that it in turn can be reduced to 2,3-octanediol (28).

Aldehyde metabolism disorders are involved in the occurrence and development of various diseases. α-l-Galactopyranose is a carbohydrate metabolized by hexose (glucose) and cyclic sugar. L-Galactose, also known as α-L-galactose or L-galactopyranose, belongs to the class of organic compounds known as hexoses. These are monosaccharides in which the sugar unit is a is a six-carbon containing moeity. L-Galactose is a primary metabolite. Primary metabolites are metabolically or physiologically essential metabolites. They are directly involved in an organism’s growth, development or reproduction. Based on a literature review very few articles have been published on L-Galactose. L-Galactose can be metabolized into vitamins.This may be related to the supplementary food provided to pregnant women in the ND group during delivery.

**Figure 2:**
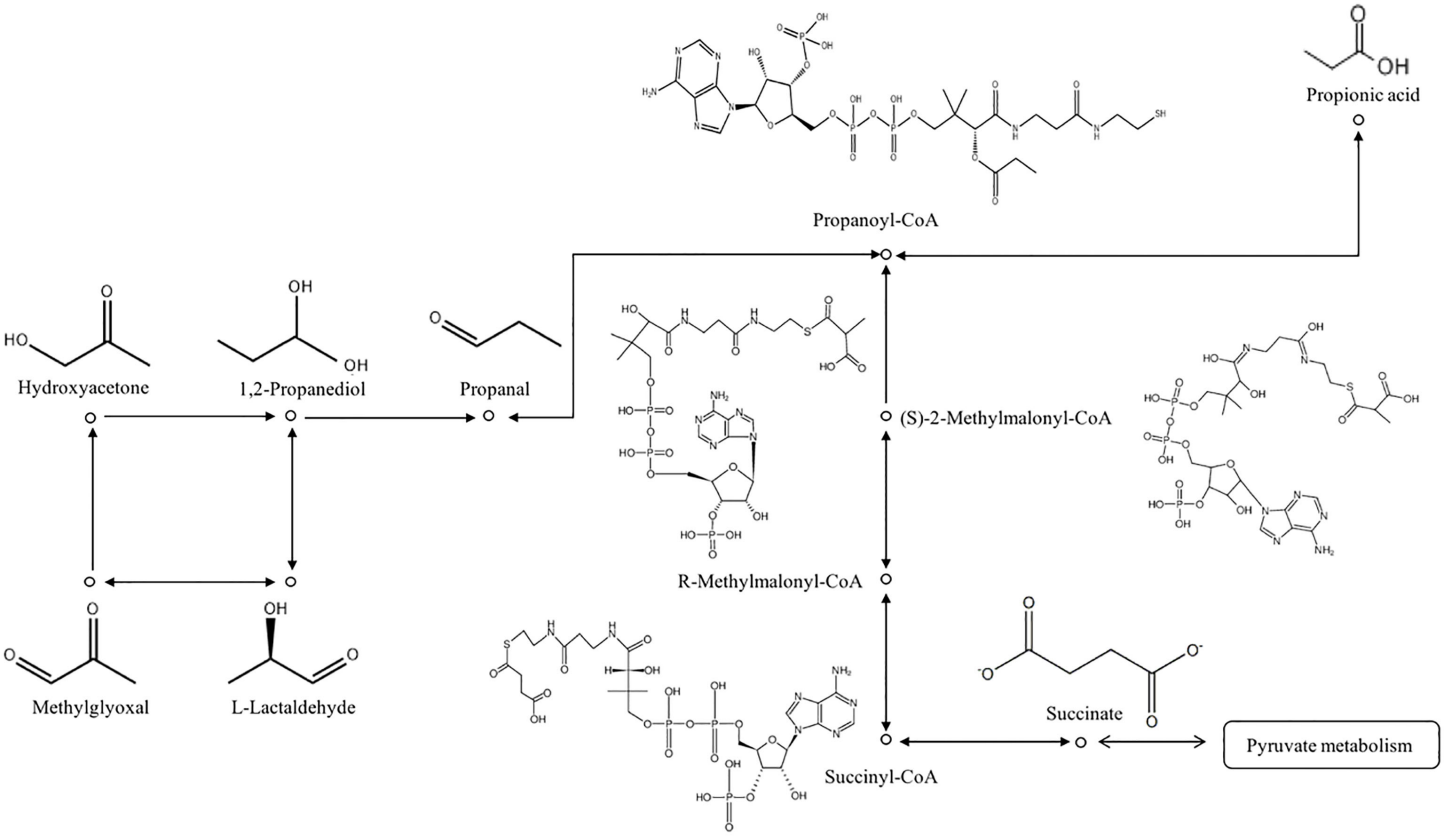
Metabolic pathway of propionic acid.

Propionic acid was identified as an important component in this study. Three different biochemical pathways for propionic acid production are succinic acid, acrylate, and propylene glycol. Propionic acid is the main end-product of succinic acid fermentation. The abundance of Bacteroides and Parabacteroides, the main producers of succinic acid, increased due to a high-fat diet and were positively correlated with body weight. Succinic acid produced by *Bacteroides thetaiotaomicron* supports the growth of *Phascolarctobacterium* and the accompanying production of propionic acid through the succinic acid pathway (29). High concentrations of *Phascolarctobacterium* have been reported in severe depression, Alzheimer’s disease, autism, and other diseases, although the heterogeneity within the disease group was also high. Therefore, the increase in propionic acid was related to the tricarboxylic acid cycle, and the metabolism of propionic acid was also related to MGO (**Figure 2**). A previous study described a metabolic pathway in athletes after marathon competition. *Veillonella* metabolizes exercise-induced lactic acid into propionic acid, which improves running time, determines a natural and microbial-encoded enzyme process, and improves sports performance (30). The delivery process is also relatively long, lasting for several hours or even longer, and the intensity is high. In pregnant women with GDM, increased glycolysis produces more pyruvate that is metabolized to lactic acid. Lactic acid is likely to be metabolized into propionic acid in the body. According to this reasoning, pyruvate can also be converted to acetone to produce MGO and aggravate nerve injury. Therefore, propionic acid may be the product of metabolic disorders in the ND group, or it may be a potential marker of cognitive dysfunction in pregnant women with GDM aggravated by labor pain. Epidural analgesia can prevent this type of injury.Malonic acid is also a kind of propionate, which may have the same metabolic pathway as propionic acid (31).

Six substances were found in the PD group. These included decane, heptane, and other alkanes. Alkanes were also observed in the ND group. Lipid peroxidation is a pathophysiological change in various diseases, including cancer, inflammatory diseases, atherosclerosis, and aging (32). Alkane metabolism is unrelated to branched-chain hydrocarbons and is the product of lipid peroxidation. Unbound alkane hydrocarbons eventually appear in the blood, urine, and exhalate. Ethane and pentane are saturated hydrocarbons produced by lipid peroxidation chain reactions (33). Therefore, these aliphatic hydrocarbons are considered biomarkers of lipid peroxidation both *in vivo* and *in vitro* (34). This indicates that pregnant women with GDM may have an oxidative stress reaction.

This study found that both groups of pregnant women had a certain degree of stress response, but the metabolic disorder of pregnant women in the ND group was more obvious, indicating that labor pain caused an increase in oxidative stress and metabolic disorders in pregnant women with GDM. In contrast, epidural analgesia can significantly improve oxidative stress metabolites in pregnant women with GDM. This is also one of the main reasons for the changes in metabolites between the two groups.

Severe labor pain activates the hypothalamus pituitary adrenal axis and sympathetic adrenal medullary axis, causing a series of neuroendocrine disorders, which also promote the secretion of glucocorticoids and catecholamines. This stress also affects the body’s immune system, thus activating inflammatory factors and oxidative stress responses, including an increase in MGO. Neuroendocrine dysfunction is one of the pathophysiological mechanisms of GDM. In the process of delivery, epidural analgesia reduces the neuroendocrine imbalance in pregnant women with GDM, adjusts the immune response, and reduces the damage caused by stress trauma to the body.

There were several limitations in this study. The sample size was relatively small. Postpartum follow-up was not performed. Finally, the duration of labor was not considered. It is reported that decane, heptane, and other alkanes are found in patients with temporary respiratory syndrome (35). Because GDM pregnant women are relatively obese, and sleep disorders in the late pregnancy may also be one of the reasons for these substances. However, these substances may also be part of the experimental reagent (such as the kit), so the kit can also release these substances during the research process, which needs further research.

## 5 Conclusion

The data demonstrate for the first time that propionic acid, a volatile substance, may be a potential marker of cognitive dysfunction in pregnant women with GDM aggravated by labor pain. Epidural analgesia can improve the expression of MGO/inflammatory/oxidative stress factors induced by childbirth-related pain.

## Data availability statement

The original contributions presented in the study are included in the article/supplementary material. Further inquiries can be directed to the corresponding authors.

## Ethics statement

The studies involving human participants were reviewed and approved by the First Affiliated Hospital of Harbin Medical University. The patients/participants provided their written informed consent to participate in this study.

## Author contributions

SS designed the study, collected data, and wrote and revised the manuscript; YL and GC interpreted and analyzed the data; LG collected the data; EL designed the study. All authors contributed to the article and approved the submitted version.
